# Does Changing Androgen Receptor Status during Prostate Cancer Development Impact upon Cholesterol Homeostasis?

**DOI:** 10.1371/journal.pone.0054007

**Published:** 2013-01-08

**Authors:** James Robert Krycer, Andrew John Brown

**Affiliations:** School of Biotechnology and Biomolecular Sciences, The University of New South Wales, Sydney, Australia; II Università di Napoli, Italy

## Abstract

**Background:**

Recent evidence associates prostate cancer with high cholesterol levels, with cholesterol being an important raw material for cell-growth. Within the cell, cholesterol homeostasis is maintained by two master transcription factors: sterol-regulatory element-binding protein 2 (SREBP-2) and liver X receptor (LXR). We previously showed that the androgen receptor, a major player in prostate cell physiology, toggles these transcription factors to promote cholesterol accumulation. Given that prostate cancer therapy targets the androgen receptor, selecting for cells with altered androgen receptor activity, how would this affect SREBP-2 and LXR activity? Using a novel prostate cancer progression model, we explored how this crosstalk between the androgen receptor and cholesterol homeostasis changes during prostate cancer development.

**Methodology/Principal Findings:**

Firstly, we characterised our progression model, which involved 1) culturing LNCaP cells at physiological testosterone levels to generate androgen-tolerant LNCaP-305 cells, and 2) culturing LNCaP-305 with the anti-androgen casodex to generate castration-resistant LNCaP-364 cells. This progression was accompanied by upregulated androgen receptor expression, typically seen clinically, and a reduction in androgen receptor activity. Although this influenced how SREBP-2 and LXR target genes responded to androgen treatment, cellular cholesterol levels and their response to changing sterol status was similar in all LNCaP sub-lines.

**Conclusion/Significance:**

Overall cholesterol homeostasis is unaffected by changing androgen receptor activity in prostate cancer cells. This does not negate the relationship between androgens and cholesterol homeostasis, but rather suggests that other factors compensate for altered androgen receptor activity. Given that cholesterol regulation is maintained during progression, this supports the growing idea that cholesterol metabolism is a suitable target for prostate cancer.

## Introduction

Since the discovery of androgens, these hormones have been closely associated with the prostate. Normal prostate cells depend upon androgens for proliferation, differentiation, and maintaining secretory functions. This is mediated by the androgen receptor (AR), the transcription factor activated by these hormones.

This concept of hormone dependence was established by Nobel laureate Charles Huggins [1], and forms the biological rationale for androgen-deprivation therapy (ADT), a major contemporary treatment strategy for metastatic prostate cancer (PCa). ADT involves medical castration to lower blood-androgen levels (from ∼10 nM testosterone [2]–[3] to optimally ∼0.7 nM) [4]. This is often supplemented by treatment with anti-androgens (e.g., casodex/biculatimide), which compete with any remaining androgens for the AR, thus aiming to completely inhibit AR function. Together, this two-part treatment is known as ‘combined androgen blockade’ [5]. Although 80–90% of patients initially respond well to ADT, the PCa eventually relapses within a median period of 18 months [6], progressing to a ‘castration-resistant’ state that renders ADT ineffective.

Castration-resistant PCa (CR-PCa) is highly-aggressive, associated with the highest mortality rates from PCa. Thus, there is a need to better understand the phenotypic changes that occur during progression to CR-PCa. One such characteristic recently gaining interest is cholesterol metabolism (e.g., [7]). High cholesterol levels have been linked with PCa risk in epidemiological studies [8]–[9], whilst laboratory studies have identified that intracellular cholesterol levels rise when prostate cells are cancerous [10]. Such cholesterol accumulation could promote PCa development as a precursor for synthesising membranes, androgens, and other players in signalling pathways [7], [11]. Thus, cholesterol-lowering drugs have been considered for PCa treatment [7]–[9], [12]. These efforts would be enhanced by studying the underlying causes of cholesterol accumulation in PCa.

Within the cell, cholesterol levels are largely regulated by two master transcription factors: sterol regulatory element-binding protein isoform 2 (SREBP-2) and liver X receptor (LXR). SREBP-2 upregulates genes involved in cholesterol synthesis (e.g., *HMGCR*) and uptake (e.g., low-density lipoprotein receptor, *LDLR*). This increases cholesterol levels, which reduces SREBP-2 activity by feedback regulation. In contrast, oxygenated cholesterol derivatives (oxysterols) activate LXR, which lowers cellular cholesterol levels by upregulating genes involved in cholesterol efflux, such as ATP-binding cassette transporter isoforms A1 (*ABCA1*) and G1 (*ABCG1*).

These two transcription factors are influenced by androgens: the AR activates SREBP-2 by upregulating its regulator, Scap [13]–[14], and inhibits LXR by coactivator competition [14]. In doing so, the AR adjusts cholesterol homeostasis in a concerted fashion, providing a mechanism for how androgens promote cholesterol accumulation in prostate cells (e.g., [15]). Given that CR-PCa arises from altering androgen (and AR) status, here we explore how cholesterol homeostasis changes during progression to CR-PCa. To achieve this, we use a novel CR-PCa progression model.

To study CR-PCa *in vitro*, progression models (e.g., [16]–[17]) are commonly generated by androgen-depriving the LNCaP cell-line, an androgen-dependent, AR-positive PCa cell-line [18]. These models are more informative than androgen-independent cell-lines, such as PC-3 which does not express AR [19], because they allow a direct comparison between the parental and androgen-independent cells.

Whilst previous LNCaP-progression models have generated a wealth of information about androgen-independent PCa (reviewed in [20]), there have been two major caveats. First, LNCaP cells are routinely cultured in media supplemented with foetal bovine serum (FBS), which contains androgen levels equivalent to a castrated human male [2]. Subsequently, the LNCaP cell-line has been selected to grow in an androgen-scarce environment, unlike clinical ‘hormone-naïve’ (pre-ADT) PCa. In fact, physiological, non-castrated testosterone levels (∼10 nM [2]–[3]) inhibit LNCaP cell growth (e.g., [21]) because the AR acts as a ‘licensing factor’ that prevents cell-cycle progression [22]–[23]. Second, LNCaP cells are typically androgen-deprived by long-term culture in media supplemented with charcoal-stripped FBS. Charcoal-stripping removes not only androgens from FBS, but other hormones and growth factors, and thus may not adequately represent clinical ADT. We have generated a progression model that overcomes these caveats [12].

Here, we characterise this model, using it to test the hypothesis that changes in AR signalling and cholesterol homeostasis in CR-PCa cells are related. Given that the AR influences cholesterol levels, examining if this interaction changes during progression to CR-PCa would help determine the potential of cholesterol metabolism as a target for CR-PCa.

## Results

### Characterisation of the castration-resistant PCa progression model

**Figure 1:**
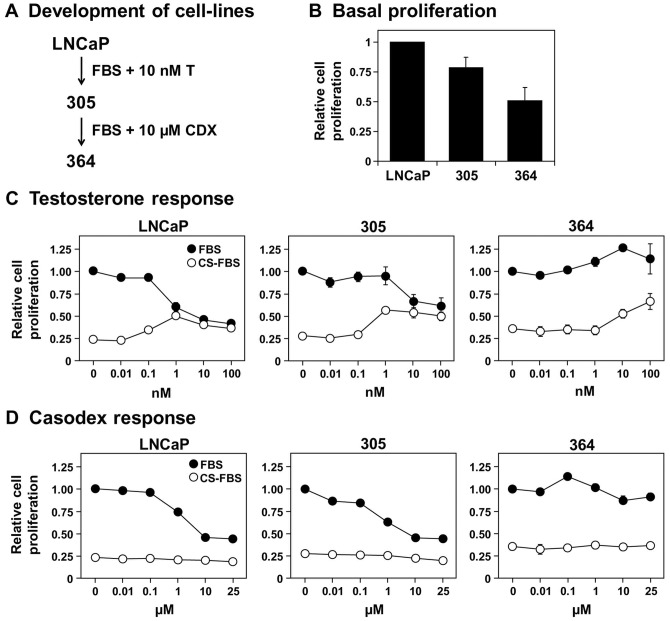
Growth characteristics of the 305 and 364 cell-lines. (A) Schematic outlining the development of these LNCaP sub-lines, involving long-term culturing in the presence of either testosterone (T) or casodex (CDX). Details in the text. (B–D) Cells were treated with 10% (v/v) sera and the concentrations of drugs indicated. In (B), this includes FBS (LNCaP) or FBS supplemented with 10 nM T (305) or 10 μM CDX (364). In (C) and (D), this includes FBS or CS-FBS, with T and CDX at concentrations indicated. Cell proliferation was determined as described in the Materials and Methods. (B–D) Data presented as mean + S.E., from three separate experiments per cell-line, each performed with quadruplicate wells per condition. In (D), error bars are contained within the symbols.

LNCaP cells were initially cultured in FBS supplemented with a physiological concentration of testosterone, generating the 305 cell-line (Figure 1A). These cells represent androgen-dependent PCa cells that can grow at serum-androgen levels, tolerating higher androgen concentrations than the parental LNCaP cells (Figure 1C). This approach for developing androgen-tolerant cells was similarly performed previously [17], except we replaced synthetic AR agonist R1881 with testosterone. It is likely that the influence of testosterone is due to direct activation of the AR or conversion to the potent androgen, dihydrotestosterone, rather than aromatisation to estrogens because testosterone and dihydrotestosterone have similar effects on cell viability (Figure S1A).

To simulate ADT (specifically, combined androgen blockade), 305 cells were cultured in FBS (containing castrate levels of androgens) supplemented with the anti-androgen casodex (Figure 1A). This generated the androgen-independent 364 cell-line, which has little proliferative response to either androgens (Figure 1C) or anti-androgens (Figure 1D). This phenotype was stable for at least 10 passages (Figure S1B). Furthermore, as a positive control, PC-3 cells were similarly unresponsive to media-androgen status (Figure S1C).

Compared to 305 and parental LNCaP cells, the 364 cells grew slower (Figures 1B, S1D) and independently of media-androgen status (Figures 1C, D). This is different to other studies (Table 1), likely due to culturing in FBS rather than CS-FBS, thus ensuring that only AR activity is targeted in our long-term selection of 364 cells. Furthermore, the independence of 364 cells from media-androgen status provided little advantage in CS-FBS (Figure 1B), implying that charcoal-stripping not only removes androgens, but other growth-promoting factors. This justifies our approach for generating a castration-resistant cell-line by casodex treatment in FBS (Figure 1A).

**Table 1:** Summary of studies that generated castration-resistant PCa cells, by long-term treatment of LNCaP cells with casodex (CDX).

	Reference	[46]	[47]	[48]	[49]	[50]	This study
	Name	LNCaP-BC2	LNCaP-Bic	LNCaP- cxD2/11/12 d	LNCaP- CS10	LNCaP- CDX1-6 e	LNCaP-364
Culturing	Serum	FBS	CS-FBS	CS-FBS	CS-FBS	CS-FBS	FBS
conditions	Drug	1+2 µM CDX	10 pM R1881	1 µM CDX (2,12) or	10 µM CDX	5 µM CDX	10 nM T, then
			+1 µM CDX	0.1 µM CDX (11)			10 µM CDX
	Time a	2 mths	3 mths	<13 wks	4 mths	3 wks	2 mths
	Clonal?	No	No	Yes	No	Yes	No
*In vitro*	Vs LNCaP b	-	↑	↑	↑	↑	- (↓ in FBS)
growth	+ androgen	↕	-	N/D	↕	↓	↑ (- in FBS)
(in CS-FBS)	+ CDX	↑ f	-	↕	↑	-	- (- in FBS)
AR protein	Vs LNCaP b	↑	-	- (2,12), ↑ (11)	-	↑	↑
	Mutated	N/D	No	Yes	No	N/D	N/D
AR activity c	Vs LNCaP b	↑	↓	- (2), ↑ (11,12)	↑	↑	↓
	+ androgen	↑	↑	N/D	↑ (blunted)	↑	-
	+ CDX	↑	↓	↑	↑ g	N/D h	↑

It should be noted that the assays for growth and AR activity differed between experiments, incuding pre-treatment, androgen used, and assay duration. N/D, not determined; ↑, increase; ↓ decrease; ↕, biphasic response; -, no effect.

a Time during CDX treatment.

b Or in comparison to parental LNCaP-104S cells for reference [50]. In our study, comparison to LNCaP or 305 cells yielded the same trends.

c This includes luciferase assays or target gene (e.g., *PSA*) mRNA and protein levels. In cases where there was a discrepancy between assays, luciferase assay results were given priority, followed by target gene mRNA levels and finally target gene protein levels.

d Parenthesised numbers below refer to the clone number (2, 11 or 12).

e These cells were derived from LNCaP-104S cells [17] rather than LNCaP cells.

f CDX treatment reduces androgen-induced proliferation, but above 1 µM CDX, growth is increased in a CDX-dose-dependent fashion independently of androgens.

g CDX increased nuclear localisation of AR.

h The study showed that CDX was not agonistic, but did not show it was antagonistic.

**Figure 2:**
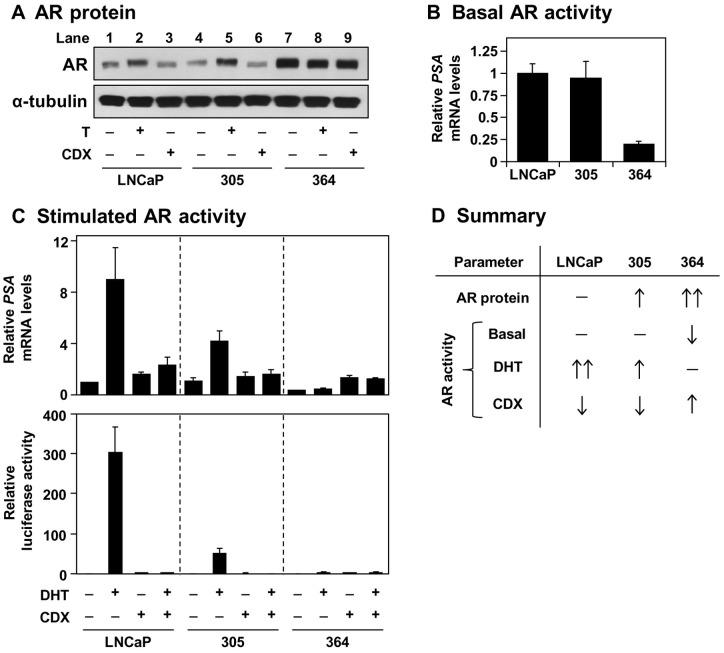
Androgen receptor status of the 305 and 364 cell-lines. (A–B) Cells were grown in Medium A with 10 nM testosterone (T) or 10 μM casodex (CDX). (A) Protein was harvested and subjected to SDS-PAGE and Western blotting against the androgen receptor (AR) and α-tubulin. (B) RNA was harvested and *PSA* mRNA levels were determined by qRT-PCR, normalised to the LNCaP cells. (C) *Top panel*: Cells were starved in Medium B for 24 h, before treatment with 1 nM dihydrotestosterone (DHT) and/or 10 μM CDX in Medium B for another 24 h. Following treatment, RNA was harvested and *PSA* mRNA levels were determined by qRT-PCR, normalised to the vehicle-treated LNCaP cells. *Bottom panel*: Following transfection, cells were seeded in Medium B. The next day, cells were treated with 1 nM DHT and/or 10 μM CDX in Medium B for another 24 h. Following treatment, cells were assayed for luciferase activity, made relative to the vehicle condition within each cell-line. (D) Summary of the results obtained in (A–C). (A) Blots are representative of four separate experiments. (B–C) Data presented as mean + S.E., from three separate experiments per cell-line, each performed with triplicate wells per condition.

Next, we characterised the AR status of these cell-lines via AR protein expression and AR activity, the latter assessed by mRNA expression of the canonical AR-target gene, *PSA* (prostate specific antigen). The autoregulation of AR levels (e.g., [24]) can be seen with testosterone and casodex treatment in LNCaP cells (Figure 2A, *lanes* 1–3). In comparison to these parental cells, 305 cells had higher AR protein levels in their basal media (Figure 2A, *lane* 5 vs 1) but similar AR activity (Figure 2B). Likewise, under androgen-deficient conditions (CS-FBS), 305 cells had a reduced serum response to dihydrotestosterone (Figure 2C), shown by both *PSA* mRNA levels (*top panel*) and *PSA* promoter activity (*bottom panel*). Together, this reflects their adaptation to higher serum-androgen levels by reduced AR activity.

Furthermore, 364 cells have even higher AR levels (Figure 2A), which has been observed in other *in vitro* studies (Table 1) and many clinical CR-PCa samples (e.g., ∼30% of clinical CR-PCa samples have AR gene amplification which has been shown to increase AR expression [25]–[27]). Although basal AR activity was lower (Figure 2B), casodex acted agonistically (Figure 2C) as seen in other studies (Table 1). Collectively, our model reflects a sizeable subset of CR-PCa and shows a change in AR activity during the progression to castration-resistance (Figure 2D).

### Does cholesterol homeostasis change in this model?

**Figure 3:**
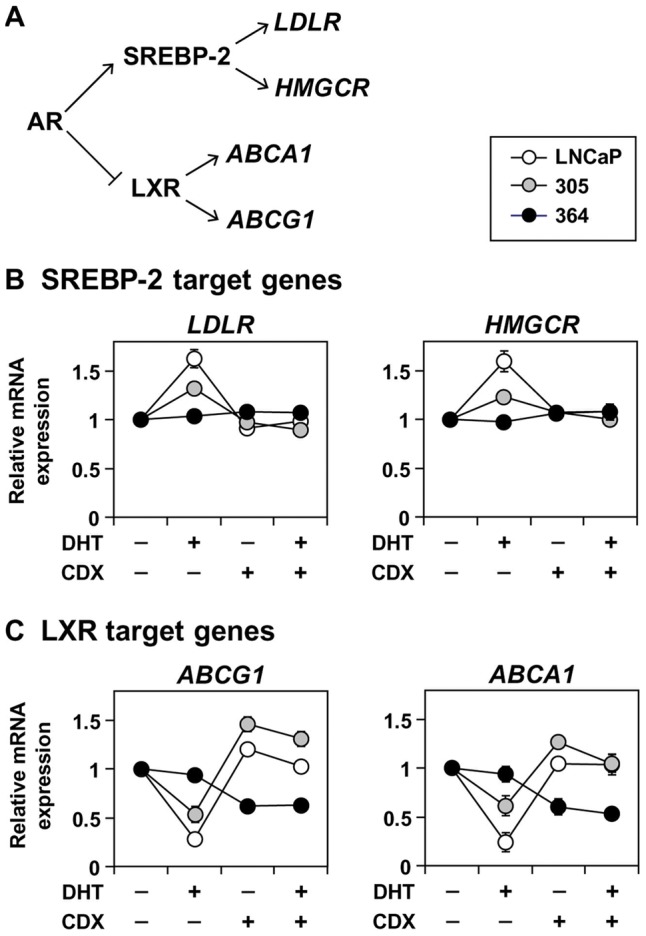
The effect of androgen receptor status on androgen-regulated cholesterol homeostasis. (A) Schematic outlining the effects of the androgen receptor (AR) on key transcription factors in cholesterol homeostasis. Details in the text. (B–C) Cells were starved in Medium B for 24 h, before treatment with 1 nM dihydrotestosterone (DHT) and/or 10 μM CDX in Medium B for another 24 h. Following treatment, RNA was harvested and (B) *LDLR* and *HMGCR*, and (C) *ABCG1* and *ABCA1*, mRNA levels were determined by qRT-PCR, normalised to the vehicle condition in each cell-line. (B–C) Data presented as mean ± S.E., from three separate experiments per cell-line, each performed with triplicate wells per condition.

Given that the AR promotes SREBP-2 activation and inhibits LXR [13]–[14] (Figure 3A), how are these interactions affected by castration-resistance? To explore this, we examined the response of SREBP-2 and LXR target genes (Figure 3A) to androgen manipulation.

As we have shown previously [14], dihydrotestosterone treatment increased SREBP-2 target gene expression (Figure 3B) and reduced LXR target gene expression (Figure 3C) in LNCaP cells, and these effects were reversed by casodex. The 305 cells exhibited a similar but blunted trend (Figures 3B, C), in line with reduced AR activity compared to LNCaP cells (Figure 2). Likewise, in 364 cells, SREBP-2 and LXR activity was unresponsive to dihydrotestosterone treatment and casodex acted agonistically to reduce LXR target gene expression (Figures 3B, C). Thus, throughout the progression model, androgen status has a varying effect on SREBP-2 and LXR.

**Figure 4:**
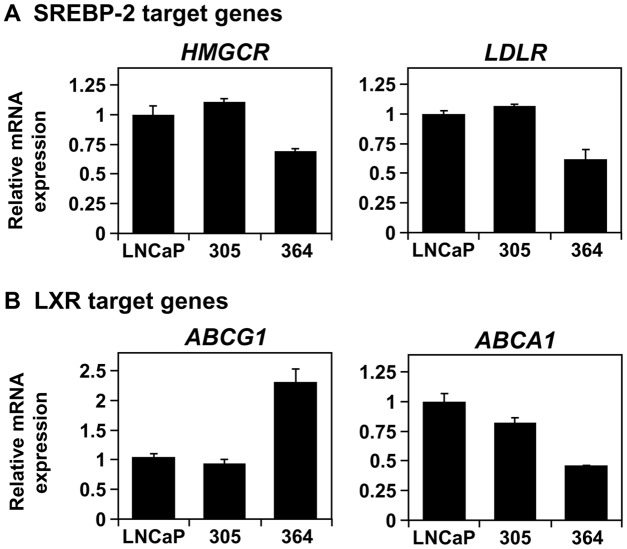
The effect of androgen receptor status on basal cholesterol homeostasis. Cells were grown in their basal media: Medium A (LNCaP), supplemented with 10 nM testosterone (305) or 10 μM casodex (364). RNA was harvested and (A) *LDLR* and *HMGCR*, and (B) *ABCG1* and *ABCA1*, mRNA levels were determined by qRT-PCR, normalised to the LNCaP cells. (A–B) Data presented as mean + S.E., from three separate experiments per cell-line, each performed with triplicate wells per condition.

Consequently, these two major cholesterol regulators should be influenced by the changing AR activity during progression. Indeed, we find a similar pattern to *PSA* expression. Firstly, there was little difference in target gene expression between LNCaP and 305 cells (Figures 4). Secondly, like *PSA*, SREBP-2 target gene expression is reduced in 364 cells (Figure 4A). Given AR antagonises LXR (Figure 3C), *ABCG1* expression is higher in 364 cells as expected, but *ABCA1* expression is reduced (Figure 4B).

**Figure 5:**
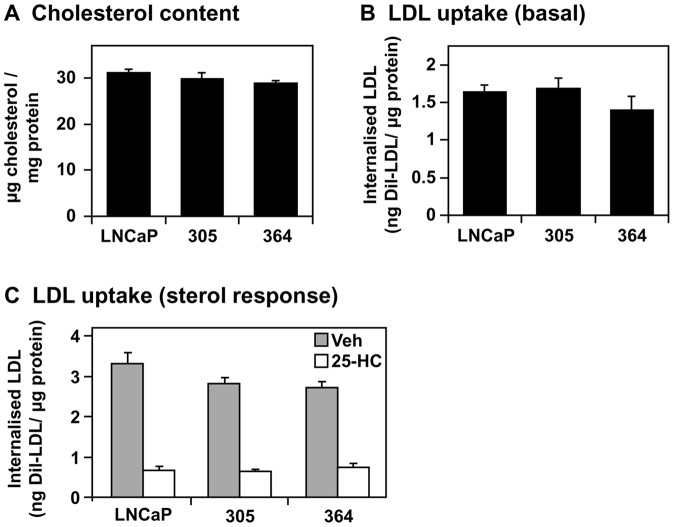
The effect of androgen receptor status on cholesterol levels and LDL uptake. (A) Cells were grown in their basal media: Medium A (LNCaP), supplemented with 10 nM testosterone (305) or 10 μM casodex (364). Cholesterol levels were determined as described in the Materials and Methods. (B) Cells were treated in their basal media, after which LDL uptake was determined. (C) Cells were plated in their basal media, then starved overnight in Medium C. The next days, cells were treated for 6 h with or without 10 μM 25-hydroxycholesterol (25-HC) in Medium C, after which LDL uptake was determined. (A–C) Data presented as mean + S.E., from three separate experiments per cell-line, each performed with triplicate wells per condition.

Despite these changes, steady state cholesterol levels were similar between the LNCaP, 305, and 364 cells (Figure 5A). Of this, ∼95% was free cholesterol (as found previously in LNCaP cells [14]) in all cell-lines (data not shown). Particularly given that there is a two-fold increase in cholesterol levels when prostate epithelial cells develop into PCa [10], this suggests that basal cholesterol homeostasis is maintained despite altered AR status during progression to castration-resistance.

**Figure 6:**
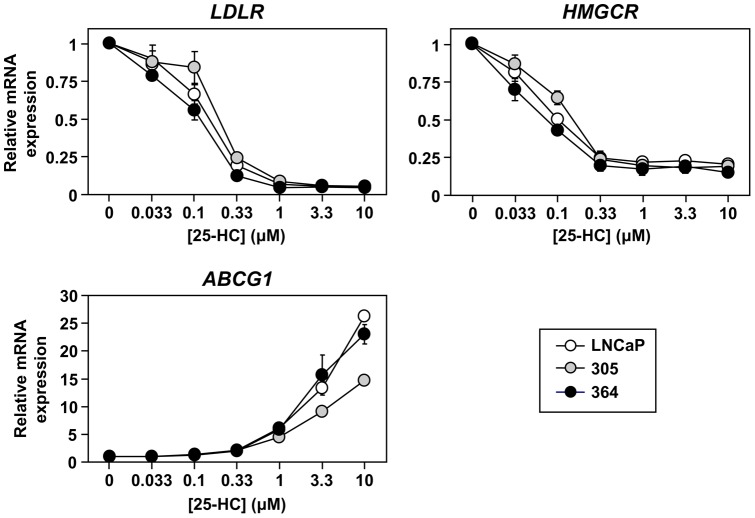
The effect of androgen receptor status on the response to changing sterol status. Cells were plated in their basal media: Medium A (LNCaP), supplemented with 10 nM testosterone (305) or 10 μM casodex (364). Cells were starved overnight in Medium C, and then treated for 6 h with 25-hydroxycholesterol (25-HC) in Medium C, at the concentrations indicated. Following treatment, RNA was harvested and *LDLR*, *HMGCR*, and *ABCG1* levels were determined by qRT-PCR, normalised to the vehicle condition within each cell-line. Data presented as mean ± S.E., from three separate experiments per cell-line, each performed with triplicate wells per condition.

However, this snapshot does not shed light on whether castration-resistant cells respond differently to changing sterol status. For instance, upon finding basal LDL uptake was similar between the cell-lines (Figure 5B), we examined the response to the oxysterol, 25-hydroxcholesterol, which reduces SREBP-2 activity and thus LDLR activity [28]. All cell-lines responded similarly to 25-hydroxycholesterol treatment (Figure 5C). To examine their sterol response further, we returned to transcriptional regulation, using 25-hydroxycholesterol to simultaneously inhibit SREBP-2 and activate LXR. While we used luciferase assays previously for this purpose [12], we analysed target gene expression here to enable us: 1) to examine LXR and SREBP-2 activity concurrently in the same cell populations, and 2) to have shorter treatment times (mRNA levels typically respond faster than promoter-driven luciferase levels), allowing us to examine an acute response to sterols. As a proof of principle, this assay confirmed that PC-3 cells have higher SREBP-2 activity than LNCaP cells (Figure S2A), as shown previously [28]. In contrast, SREBP-2 responded similarly to 25-hydroxycholesterol in LNCaP, 305, and 364 cells (Figure 6, *top panels*).

In addition, this oxysterol had the same effect on LXR-target gene (*ABCG1*) expression in both LNCaP and 364 cells (Figure 6, *bottom panel*). This response was weaker in 305 cells, but recovered when the cells were seeded in FBS without testosterone supplementation (Figure S2B). Tangentially, we found a similar effect in LNCaP cells (data not shown), suggesting that these cells can accumulate androgens preceding treatment in unsupplemented media; this residual androgen could in turn stimulate AR and inhibit LXR-driven *ABCG1* expression (Figure 3C). Nevertheless, this model suggests that blunted AR activity in castration-resistant cells does not impact upon their ability to respond to cellular sterol status.

## Discussion

In this study, we characterise an *in vitro* PCa model that consists of three components (Figure 1): (1) LNCaP cells, which are adapted to low serum-androgen levels, (2) 305 cells, adapted to high serum-androgen levels, and (3) 364 cells, adapted to growth independent of serum-androgen status. These adaptations were accompanied by changes in AR activity (Figure 2). Although the cross-talk between AR and cholesterol regulation diminishes from LNCaP to 305 to 364 cells (Figure 3), we found that overall cholesterol homeostasis remains unaffected (Figures 4,5,6).

In our PCa model, the androgen-tolerant 305 cells had reduced AR responsiveness compared to LNCaP cells (Figure 2), but were equally sensitive to androgen-deprivation (Figure 1). Thus, we simulated combined androgen blockade by casodex treatment in androgen-poor FBS. The resulting 364 cells have higher AR expression, which is a consistent change with progression to CR-PCa *in vivo* [29] and observed in clinical samples [25], [30]. Increased AR expression can cause anti-androgens to act as agonists (e.g. [16], [29], [31]), as observed here (Figure 2) – this relies on the AF-1 domain of AR, suggesting altered stoichiometry with transcriptional coregulators [31]. Increased AR expression and casodex agonism is a common theme shared with past studies (Table 1) – interestingly, CS-FBS was used in these studies (Table 1, Figure 2), whilst this casodex agonism was lost in FBS (data not shown), suggesting the serum background may influence the coregulator stoichiometry. Nevertheless, by exploiting the low androgen content of FBS and avoiding long-term culture in CS-FBS, we have obtained three LNCaP sub-lines that vary in their AR activity.

Given the crosstalk between AR, SREBP-2 and LXR (Figure 3), these cells provide the opportunity to examine the effect of differing AR states in PCa on cholesterol homeostasis (Figures 4–5). From the LXR axis, we observed a divergent response between the LXR gene targets: with reduced AR activity in 364 cells (Figure 2), basal *ABCG1* expression expectedly rose, whilst *ABCA1* expression was reduced (Figure 4). This was observed before in castration-resistant cells selected from long-term culturing in CS-FBS [32], suggesting that the AR may also influence ABCA1 through an intermediate that opposes LXR. This same intermediate may also influence SR-BI, another LXR target gene (e.g., [33]) which mediates HDL uptake and efflux [34], since *SR-BI* mRNA expression was also reduced in 364 cells (Figure S3). Furthermore, preliminary experiments showed no differences in serum-dependent cholesterol efflux between our LNCaP sub-lines (data not shown), but future studies should consider specific cholesterol carriers to dissect the exchange of cholesterol with the extracellular environment, in order to determine the consequences of this divergent regulation between SR-BI, ABCA1, and ABCG1.

In contrast, another group cultured LNCaP xenografts in castrated mice, finding that *ABCA1* and *SR-BI* expression was increased in the castration-resistant tumours [35], but *ABCG1* expression was not investigated. Using this same model, *SREBP-2* mRNA and activated SREBP-2 protein were higher following progression to CR-PCa [36], along with increased cholesterol synthesis [35]. This correlates with expression profile studies in patients, finding increased expression of SREBP-2 [37] and sterol biosynthetic [38] genes. Another study found decreased expression [39], in line with the decline in SREBP-2 target genes observed here in 364 cells, which in turn correlates with reduced AR activity. Despite these changes, steady state cholesterol levels were similar between androgen-dependent PCa and CR-PCa cells, both in this study (Figure 5A) and in the *in vivo* xenograft model [35]. To the best of our knowledge, there is no information on the cholesterol levels of CR-PCa metastases.

However, these observations do not account for the dynamic nature of cholesterol homeostasis. Thus, we examined the effect of AR status on the responsiveness of cells to changing sterol status – as far as we are aware, this is the first study to compare such cholesterol homeostasis between parental and castration-resistant LNCaP cells. Whilst SREBP-2 is normally feedback-regulated by sterols, evidence suggests that CR-PCa cells are sterol-resistant, based on higher mature SREBP-2 *in vivo* [36] and higher SREBP-2 activity in AR-negative PC-3 cells compared to LNCaP cells ([28], Figure S2A). However, we found that SREBP-2 and LXR in LNCaP, 305, and 364 cells responded similarly to sterols (Figure 6), with similar results at the functional level with LDL uptake (Figure 5). Being circumspect, this progression model involves long-term culture which may produce changes other than AR status. Thus, one could argue that these findings could be supported by more direct genetic manipulations (e.g., AR overexpression and knockdown), but increasing AR expression may not necessarily enhance AR activity (as seen in 364 cells), transient transfections would influence viability (similarly to manipulating AR activity in Figure 1), and stable transfections would involve long-term culture and thus experience the same caveats.

Nevertheless, the findings here imply that there are compensatory mechanisms to counteract any changes in cholesterol homeostasis [40], due to loss of basal AR activity. For instance, androgen ablation is accompanied by a rise in active Akt [41], a signalling kinase which we have found to enhance SREBP-2 activation [42]. Furthermore, whilst the current *in vitro* study enables a reductionist approach and manipulations such as altering cellular sterol status, it does not account for changes occurring *in vivo*. For instance, prostate tissue is normally hypoxic, and this is enhanced by androgen ablation [43] – given the relationship between sterol homeostasis and oxygen levels [44], future experiments should explore this in PCa.

Overall, our work does not negate the relationship between androgens and cholesterol homeostasis in PCa cells (Figure 3), but suggests that other factors may compensate for the changes in basal AR activity between different PCa cells. These need to be elucidated in future experiments. If cholesterol regulation is unaltered during progression to CR-PCa, this has two implications: first, that the cells need to maintain sufficient cholesterol content to sustain growth (regulators mediating this still need to be found), and second, that CR-PCa would be equally susceptible as naïve PCa to drugs that manipulate cholesterol metabolism. We previously found that LNCaP and 364 cells are both sensitive to drugs that inhibit SREBP-2 activity [12], supporting the growing idea that cholesterol metabolism is a suitable target for CR-PCa [7].

## Materials and Methods

### Materials

FBS was obtained from Bovogen (Vic, AU) and penicillin/streptomycin from Life Technologies (Vic, AU). All other media components were obtained from Sigma-Aldrich (NSW, AU). As described previously, FBS was made hormone-deficient by generating charcoal-stripped FBS (CS-FBS) [14], or cholesterol-deficient by generating lipoprotein-deficient FBS (FBLPDS) [28]. DiI-labelled LDL (DiI-LDL) was prepared as described previously [28]. Casodex (bicalutamide), Hoechst-33258, compactin (mevastatin), mevalonate, and 25-hydroxycholesterol were obtained from Sigma-Aldrich. Testosterone and dihydrotestosterone were gifts from Dr David Handelsman (ANZAC Research Institute, NSW, AU).

### Cell culture

The PCa cell-lines, LNCaP [18] and PC-3 [19], were a gift from Dr Pamela Russell (Australian Prostate Cancer Research Centre, Qld, AU), and were maintained in Medium A (RPMI 1640, supplemented with 10% (v/v) FBS, 100 U/ml penicillin and 100 μg/ml streptomycin). The generation of LNCaP-305 (‘305’) and LNCaP-364 (‘364’) cells was described previously [12]. The numbers ‘305’ and ‘364’ indicate the experiment index number. 305 and 364 cells were maintained and seeded in their selection media, being Medium A supplemented with 10 nM testosterone or 10 μM casodex respectively. Prior to plating cells, plates and dishes were treated with polyethyleneimine (Sigma-Aldrich) to enhance cellular adhesion as described previously [12]. As specified in experiments, PCa cells were treated in Medium B (RPMI 1640, supplemented with 10% (v/v) CS-FBS, 100 U/ml penicillin and 100 μg/ml streptomycin) to remove the influence of exogenous androgens. Alternatively, cells were treated in Medium C (RPMI, supplemented with 10% (v/v) FBLPDS, 5 μM compactin, 50 μM mevalonate, 100 U/ml penicillin and 100 μg/ml streptomycin) to lower cellular cholesterol status for subsequent sterol treatment [12].

### Hoechst assay for cell proliferation

Cells were seeded and treated as in cell viability assays previously described by our group [12] – briefly, cells were seeded at 10,000 cells per well in 96-well plates in phenol-red-free RPMI, supplemented with 0.1% (v/v) bovine serum albumin. The next day, cells were treated by addition of an equal volume of phenol-red-free RPMI containing serum and drugs to each well, to achieve the final concentrations specified in the figures. Following treatment, the WST-1 assay was avoided here because we found that higher androgen concentrations stimulated WST-1 reduction whilst reducing cell viability (Figure SA), thus dissociating the assumed correlation between metabolic activity and viability in the WST-1 assay. Thus, cell growth was instead quantified using a Hoechst stain assay [45], with some modifications: The media was aspirated and the plate was frozen at −80°C. Plates were briefly thawed at room temperature and 100 μl water added per well before freezing again at −80°C. Plates were thawed, followed by addition of 100 μl Hoechst-33258 solution, containing 10 μg/ml Hoechst-33258 in TNE buffer (10 mM Tris-HCl pH 7.4, 2 M NaCl, 1 mM EDTA). The fluorescence was measured at *F*_Ex_  = 360 nm and *F*_Em_  = 440 nm, using the Fluostar Galaxy fluorometer (BMG Labtech, Vic, AU).

### Western blotting

Following treatment, cellular protein was analysed by Western blotting as described previously [14] – briefly, cells were lysed using SDS ((1% [w/v] SDS, 10 mM Tris-HCl [pH 7.6], 100 mM NaCl), supplemented with 2% (v/v) protease-inhibitor cocktail (Sigma-Aldrich)) and protein content determined using the Pierce BCA Protein Assay (Thermo Fisher Scientific, Vic, AU). Protein aliquots (50 μg) were subjected to 7.5% (w/v) SDS-PAGE, and transferred to Trans-Blot transfer medium (Bio-Rad, Regents Park, NSW, AU). Membranes were probed with the following various antibodies: anti-α-tubulin (mouse clone B-5-1-2, from Sigma-Aldrich) and anti-AR (rabbit clone, catalog #3202 from Cell Signaling Technology, MA, USA). Antibodies were visualised on Hyperfilm (GE Healthcare, NSW, AU) using the ECL detection system (Millipore, NSW, AU).

### Quantitative real-time reverse-transcription PCR (qRT-PCR)

Following treatment, mRNA levels were determined as described previously [14] – briefly, RNA was harvested using Trizol (Life Technologies) and reverse-transcribed using the SuperScript III First-Strand Synthesis System (Life Technologies), and expression levels of target genes were quantified by qRT-PCR, using the SensiMix SYBR No-ROX kit (Bioline, NSW, AU) and Rotorgene-Q (Qiagen, Vic, AU). Primers have been described previously for human *PBGD*, *LDLR*, and *HMGCR* [14], as well as *ABCG1* [12]. In addition, *ABCA1* primer sequences were provided by Dr. Etienne Lefai (Faculté de Médecine Lyon Sud, Lyon, FR) and *SR-BI* primer sequences were hSR-BI-F (5′-ACAAGTGGAACGGGCTGA-3′) and hSR-BI-R (5′-AGAACTCCAGCGAGGACTCA-3′).

### Luciferase reporter assay

The reporter plasmid, PSA-luc, contains firefly luciferase driven by the *PSA* promoter, and was a gift from Dr Hong Wu Chen (Davis Cancer Centre, University of California, US). The luciferase assay was performed as described previously [14] – briefly, cells were transfected in 60 mm dishes using *Trans*IT-2020 reagent (MirusBio, WI, USA) and then seeded into 24 well dishes in Medium B. The next day, cells were treated for 24 h prior to harvesting and assaying. Luciferase assay was measured using the Luciferase Assay System (Promega, NSW, AU), normalised to protein content, and made relative to the vehicle condition to obtain ‘relative luciferase activity’.

### Cholesterol assay

Following treatment, cells were lysed as described previously [12]. Cholesterol content was determined using the Amplex Red Cholesterol Assay Kit (Invitrogen), and normalised to protein content, which was determined using the Pierce BCA Protein Assay. As positive controls, this assay could detect a ∼40% change in total cholesterol levels with cyclodextrin treatment (A. Prabhu, J. R. Krycer, and A. J. Brown, unpublished data).

### LDL uptake assay

LDLR activity was assayed as described previously [28], with slight modifications. After seeding in 12-well plates, cells were treated with fresh media (as described in the figure legends). This was performed in duplicate wells. Both sets of cells were then incubated in 0.5 ml RPMI, supplemented with 10% (v/v) FBLPDS and 10 μg/ml DiI-LDL, for 2 h – one set of cells at 37°C and the other at 4°C. Cells were lysed in NaOH/SDS lysis buffer (0.1 M NaOH, 0.1% (w/v) SDS), and needled 15 times with a blunt 18-gauge needle. Cell lysate was assayed for DiI-LDL content by fluorescence at *F*_Ex_  = 544 nm and *F*_Em_  = 610 nm, using the Fluostar Galaxy fluorometer (BMG Labtech), and for protein content using the BCA assay kit (Pierce). DiI-LDL content was normalised to protein content for each sample. The difference between these normalised values between cells incubated at 37°C and 4°C was used to determine the internalised DiI-LDL.

## Supporting Information

Figure S1 Additional studies into the response of prostate cancer cells to varying media-androgen levels. (A) LNCaP, 305, and 364 cells were treated and assayed as in Figure 1C, with the indicated concentrations of dihydrotestosterone or testosterone. Cell proliferation was made relative to the vehicle condition (vehicle condition not shown). Data presented as mean + S.E., from three separate experiments, with quadruplicate wells per condition. (B) After the establishment of the 364 sub-line, these cells were passaged an additional 10 times before performing repeating the experiments described in Figures 1B and C. Representative of two separate experiments. Data is presented as mean ± S.D., from quadruplicate wells per condition. (C) PC-3 cells were plated and treated as described in Figures 1B and C. Data is presented as mean ± S.D., from quadruplicate wells per condition. (D) The relative cell proliferation rates of LNCaP, 305, and 364 cells in Medium A were determined as described in the Materials and Methods. Whether comparing growth in FBS (here) or basal media (Figure 1B), 364 cells proliferate slower than LNCaP and 305 cells. Data presented as mean + S.E., from three separate experiments, with quadruplicate wells per condition.
(TIF)

Figure S2 The examination of PC-3 cells and the influence of testosterone in the sterol response assay. (A) PC-3 cells were treated and analysed as described in Figure 6. Data presented as mean ± S.E. (half-range), from two separate experiments conducted with triplicate wells per condition. The LNCaP dataset was sourced from Figure 6 as a comparison. (B) 305 cells were seeded in Medium A, supplemented with (FBS/T) or without (FBS) 10 nM testosterone. Following seeding, cells were starved overnight in Medium C, then treated for 6 h with or without 10 μM 25-hydroxycholesterol (25-HC) in Medium C. Following treatment, RNA was harvested and *ABCG1* mRNA levels were determined by qRT-PCR and normalised to the vehicle FBS/T condition. Data is presented as mean + S.D., representative of two experiments performed with triplicate wells per condition.
(TIF)

Figure S3 *SR-BI* mRNA expression is reduced in 364 cells. Cells were treated as described in Figure 4. RNA was harvested and *SR-BI* mRNA levels were determined by qRT-PCR and normalised to the LNCaP cells. Data presented as mean + S.E., from three separate experiments per cell-line, each performed with triplicate wells per condition.
(TIF)

Figure S4 The WST-1 assay does not correlate with cell viability upon androgen treatment. LNCaP cells were seeded and treated in preparation for the WST-1 assay as described previously [12]. Treatment was 1 nM dihydrotestosterone (DHT) in phenol-red-free RPMI, supplemented with 10% (v/v) FBS, for 3 days. (A) Phase contrast microscopy was performed using the Olympus CKX31 microscope (Olympus, NSW, AU), with micrographs captured using the Moticam 2300 camera (Motic, Xiamen, CH). (B) The WST-1 assay was performed as described previously [12]. Data presented as mean + S.D., from quadruplicate wells per condition. Although androgen treatment reduces cell viability by visual inspection (A), the WST-1 assay does not detect this (B). In contrast, the Hoechst stain demonstrated a similar decrease in cell proliferation (Figure S1A).
(TIF)
