# Supplementary figures and images for: Does Changing Androgen Receptor Status during Prostate Cancer Development Impact upon Cholesterol Homeostasis?
Source: PLoS One. 2013 Jan 8;8(1):e54007. doi: 10.1371/journal.pone.0054007 (PMC3540066; doi:10.1371/journal.pone.0054007)

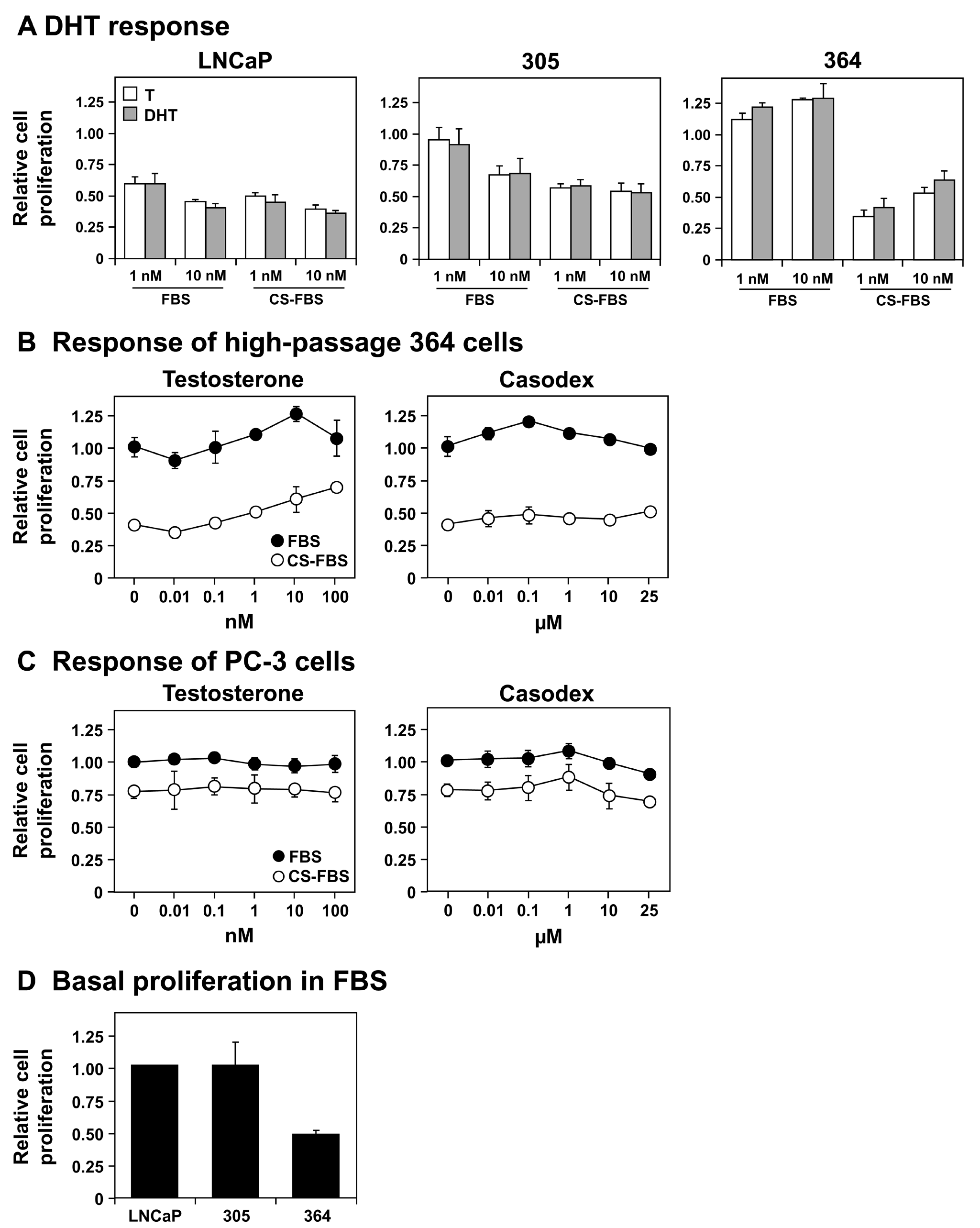

Supplement: Figure S1 — Additional studies into the response of prostate cancer cells to varying media-androgen levels. (A) LNCaP, 305, and 364 cells were treated and assayed as in Figure 1C, with the indicated concentrations of dihydrotestosterone or testosterone. Cell proliferation was made relative to the vehicle condition (vehicle condition not shown). Data presented as mean + S.E., from three separate experiments, with quadruplicate wells per condition. (B) After the establishment of the 364 sub-line, these cells were passaged an additional 10 times before performing repeating the experiments described in Figures 1B and C. Representative of two separate experiments. Data is presented as mean ± S.D., from quadruplicate wells per condition. (C) PC-3 cells were plated and treated as described in Figures 1B and C. Data is presented as mean ± S.D., from quadruplicate wells per condition. (D) The relative cell proliferation rates of LNCaP, 305, and 364 cells in Medium A were determined as described in the Materials and Methods. Whether comparing growth in FBS (here) or basal media (Figure 1B), 364 cells proliferate slower than LNCaP and 305 cells. Data presented as mean + S.E., from three separate experiments, with quadruplicate wells per condition. (TIF) [file pone.0054007.s001.tif]

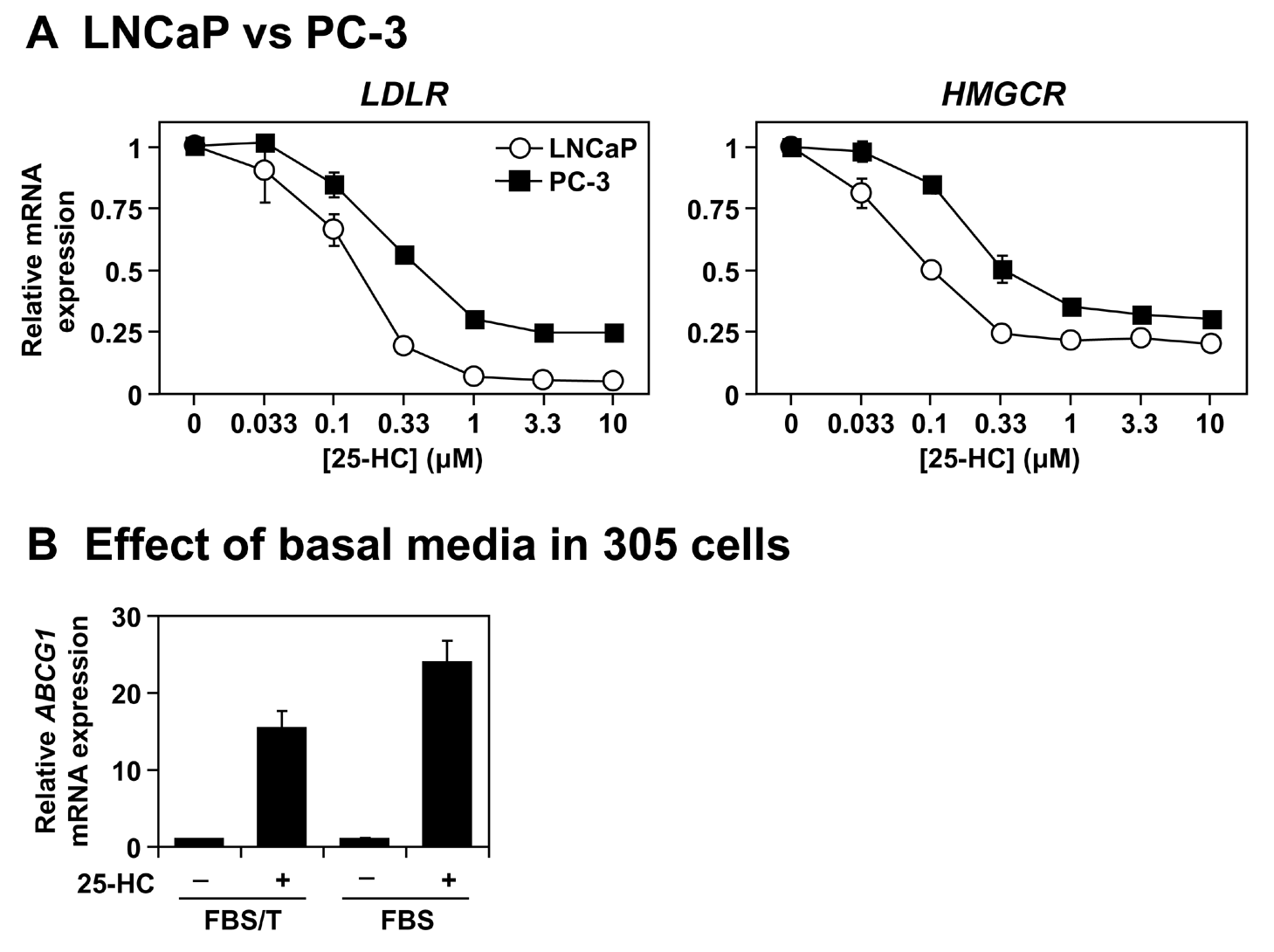

Supplement: Figure S2 — The examination of PC-3 cells and the influence of testosterone in the sterol response assay. (A) PC-3 cells were treated and analysed as described in Figure 6. Data presented as mean ± S.E. (half-range), from two separate experiments conducted with triplicate wells per condition. The LNCaP dataset was sourced from Figure 6 as a comparison. (B) 305 cells were seeded in Medium A, supplemented with (FBS/T) or without (FBS) 10 nM testosterone. Following seeding, cells were starved overnight in Medium C, then treated for 6 h with or without 10 μM 25-hydroxycholesterol (25-HC) in Medium C. Following treatment, RNA was harvested and ABCG1 mRNA levels were determined by qRT-PCR and normalised to the vehicle FBS/T condition. Data is presented as mean + S.D., representative of two experiments performed with triplicate wells per condition. (TIF) [file pone.0054007.s002.tif]

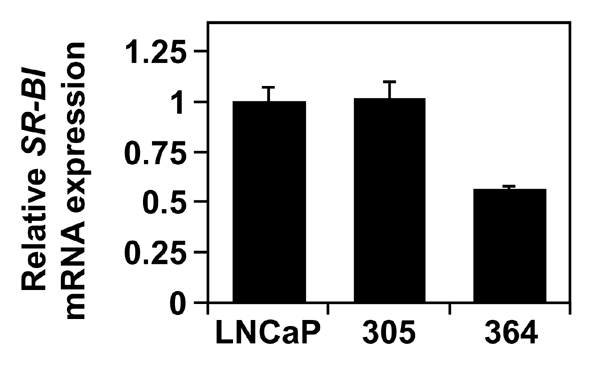

Supplement: Figure S3 — SR-BI mRNA expression is reduced in 364 cells. Cells were treated as described in Figure 4. RNA was harvested and SR-BI mRNA levels were determined by qRT-PCR and normalised to the LNCaP cells. Data presented as mean + S.E., from three separate experiments per cell-line, each performed with triplicate wells per condition. (TIF) [file pone.0054007.s003.tif]

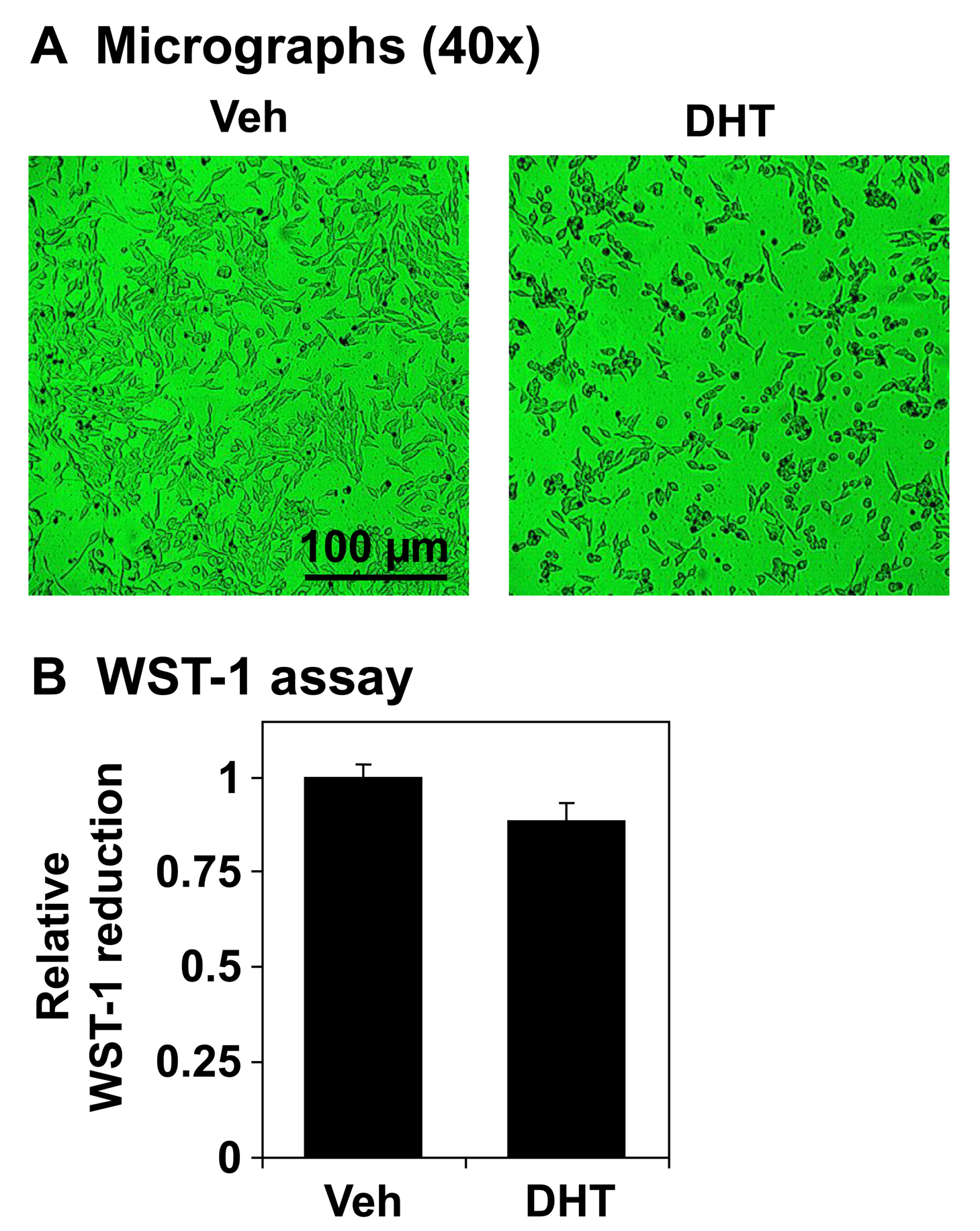

Supplement: Figure S4 — The WST-1 assay does not correlate with cell viability upon androgen treatment. LNCaP cells were seeded and treated in preparation for the WST-1 assay as described previously [12]. Treatment was 1 nM dihydrotestosterone (DHT) in phenol-red-free RPMI, supplemented with 10% (v/v) FBS, for 3 days. (A) Phase contrast microscopy was performed using the Olympus CKX31 microscope (Olympus, NSW, AU), with micrographs captured using the Moticam 2300 camera (Motic, Xiamen, CH). (B) The WST-1 assay was performed as described previously [12]. Data presented as mean + S.D., from quadruplicate wells per condition. Although androgen treatment reduces cell viability by visual inspection (A), the WST-1 assay does not detect this (B). In contrast, the Hoechst stain demonstrated a similar decrease in cell proliferation (Figure S1A). (TIF) [file pone.0054007.s004.tif]
